# Letter to the Editor regarding “Comparison of phytochemical composition of *Ginkgo biloba* extracts using a combination of non-targeted and targeted analytical approaches”

**DOI:** 10.1007/s00216-021-03690-0

**Published:** 2021-10-30

**Authors:** Mario Wurglics, Manfred Schubert-Zsilavecz

**Affiliations:** Institute of Pharmaceutical Chemistry, Goethe University, Frankfurt am Main, Germany

Herbal supplements, usually used as dry extracts, are multi-substance mixtures. For their characterization, it is essential to specify the drug-extract ratio and indicate the extraction solvent used. The National Toxicology Program (NTP) has evaluated a ginkgo-based herbal product for toxicity and carcinogenicity in short-term and long-term studies [1]. The transferability of the results to other ginkgo products plays a key role in this project. With their study, Collins and colleagues want to prove the question of the transferability of the NTP results based on the comparison of the phytochemical composition of *Ginkgo biloba* extracts (GbE) using a combination of non-targeted and targeted analytical approaches [2].

Notwithstanding the great importance of this project, we consider the conclusions drawn from the results of the pilot study to be largely speculative and scientifically insufficiently substantiated.

We base our criticism on four points in particular:

- *Extract characterization*
  The production of a standardized extract of *G. biloba* leaves is a multi-step process. The quality of the starting material and the production process decisively influence the quality and composition of the product (GbE). A minimum requirement for the comparison of herbal extracts is the indication of the drug-extract ratio (DER) and the information about which solvent was used for the extraction process.
  EGb 761® is an aqueous-acetonic dry extract (DER 35–67:1) that contains 22 to 27% flavonol glycosides, 5.4 to 6.6% terpene trilactones, and less than 5 ppm ginkgolic acids, according to the European Pharmacopoeia [3].
  For the NTP test extract, the contents of flavone glycosides, terpene trilactones, and ginkgolic acids are listed, but the DER and the extraction solvent are not described. In our opinion, it is scientifically inappropriate to use an uncharacterized ginkgo extract as a reference extract for determining extract similarities.
- *HPLC-ELSD analytics*
  A HPLC-ELSD method was used to generate chromatographic fingerprints of the studied extracts. Apart from the analytical limitations of this HPLC method, it is annoying that Figs. 1 and 2 in the publication of Collins and colleagues [2] are apparently identical and do not show the differences between the unhydrolyzed and hydrolyzed samples (in our opinion, both figures show the hydrolyzed samples). However, the chromatograms of the hydrolyzed samples clearly show that the chromatographic profile of EGb 761® differs significantly from that of the NTP test product.
- *Non-targeted NMR*
  NMR spectroscopy is an excellent and indispensable analytical tool for the characterization of (complex) molecular structures. In contrast, NMR is not very suitable for the determination of similarities in the composition of complex substance mixtures, not least because of a lack of sensitivity and resolution. In this light, the assignment of compositional similarities of GbE based on dendrogram analysis of similarities of NMR spectra appears to be not very plausible.
- *HPTLC*
  High-performance thin-layer chromatography (HPTLC) was used to compare the similarities of the studied extracts. As described for the methods above, HPTLC is also limited concerning its resolution und sensitivity. Aside from the method limitations, no comparison of HPTLC lanes of the NTP test article and EGb 761® is presented in the results of the study, and thus, this data cannot contribute to a similarity comparison of the products.

The concerns described above, about the different specifications of the GbE products and hence well-known chemical differences between the extracts, the method limitations, the apparent differences in the presented HPLC data, the lack of direct NMR and HPTLC comparability, and the lack of methods to detect important substance classes beside the flavonoids and terpene trilactones, raise the question to what extent the conclusions can be supported. The assessment of chemical profiles of herbal extracts requires profound understanding of the compounds present in the different extracts investigated and the use of multiple tailored analytical methods to detect und compare substance patterns from polymers, and of major constituents down to compounds in trace amounts.

For all the above reasons, we are more than critical of Collins’ claim to be able to easily determine a sample’s authenticity of a complex GbE using a simple analysis. Furthermore, we do not consider the methods presented to be suitable for making robust comparisons of the similarities of complex herbal extracts.

## References

[1] National Toxicology Program (2013). Toxicology and carcinogenesis studies of *Ginkgo biloba* extract (CAS No. 90045-36-6) in F344/N rats and B6C3F1/N mice (Gavage studies). Natl Toxicol Program Tech Rep Ser.

[2] Collins BJ, Kerns SP, Aillon K (2020). Comparison of phytochemical composition of *Ginkgo biloba* extracts using a combination of non-targeted and targeted analytical approaches. Anal Bioanal Chem.

[3] European pharmacopoeia 10th edition. 2020; Strasbourg: Council of Europe.

